# Preliminary Findings on a Novel Behavioural Approach for the Assessment of Pain and Analgesia in Lambs Subject to Routine Husbandry Procedures

**DOI:** 10.3390/ani10071148

**Published:** 2020-07-07

**Authors:** Emily P. Grant, Sarah L. Wickham, Fiona Anderson, Anne L. Barnes, Patricia A. Fleming, David W. Miller

**Affiliations:** 1 Agricultural Sciences, College of Science, Health, Engineering & Education, Murdoch University, Murdoch WA 6150, Australia; sarahlwickham@outlook.com (S.L.W.); F.Anderson@murdoch.edu.au (F.A.); A.Barnes@murdoch.edu.au (A.L.B.); T.Fleming@murdoch.edu.au (P.A.F.); D.Miller@murdoch.edu.au (D.W.M.); 2 Cooperative Research Centre for Sheep Industry Innovation (Sheep CRC), Armidale NSW 2350, Australia

**Keywords:** qualitative behavioural assessment (QBA), mulesing, analgesia, pain, sheep, animal welfare

## Abstract

**Simple Summary:**

The identification and assessment of pain in sheep are important but, due to their stoic nature, are difficult. In the present study, we evaluated the use of qualitative behavioural assessment to assess pain in lambs caused by routine husbandry procedures performed at lamb marking (ear tagging, castration, mulesing, and tail docking). To do this, video footage of control lambs and of lambs subject to these procedures that were either administered analgesics (Tri-Solfen and meloxicam) or a placebo, was captured 1.5 h post-procedure and assessed by 19 observers. Results showed that the observers agreed in their assessment of the lambs and, as expected, the pain caused by the husbandry procedures altered the behavioural patterns and demeanour of the lambs in a way that was captured by observers using this approach. At the time of assessment, it also appears that the analgesics administered did not reduce the pain experienced by those lambs that received them. These results suggest that qualitative behavioural assessment may be useful in identifying pain in lambs; however further work is needed to test this methodology with lambs given effective analgesic pain relief.

**Abstract:**

The identification and assessment of pain in sheep under field conditions are important, but, due to their stoic nature, are fraught with many challenges. In Australia, various husbandry procedures that are documented to cause pain are routinely performed at lamb marking, including ear tagging, castration, mulesing, and tail docking. This study evaluated the validity of a novel methodology to assess pain in lambs: qualitative behavioural assessment (QBA) was used to compare the behavioural expression of control lambs (CONTROL) with that of lambs subject to these procedures that received either a saline placebo 15 min before procedures (PLACEBO), or were administered meloxicam 15 min before procedures in addition to the standard analgesic Tri-Solfen at the time of procedures, as per the manufacturer’s recommendations (ANALGESIC TREATMENT; AT). In terms of behavioural expression, it was expected that: CONTROL ≠ PLACEBO, AT = CONTROL, and PLACEBO ≠ AT. Video footage of the 6−8-week-old lambs (*n* = 10 for each treatment) was captured approximately 1.5 h postprocedure and was presented, in a random order, to 19 observers for assessment using the Free-Choice Profiling (FCP) approach to QBA. There was significant consensus (*p* < 0.001) among the observers in their assessment of the lambs, with two main dimensions of behavioural expression explaining 69.2% of the variation. As expected, observers perceived differences in the demeanour of lambs in the first dimension, scoring all lambs subject to the routine husbandry procedures as significantly more ‘dull’ and ‘uneasy’ compared to the control lambs (*p* < 0.05). Contrary to expectations, the results also suggested that analgesic treatment did not provide relief at the time of observation. Further investigations to validate the relationship between behavioural expression scores and pain are necessary, but these results suggest that painful husbandry procedures alter the behavioural expression of lambs and these differences can be captured using QBA methodology.

## 1. Introduction

Animal species differ markedly in their observable reactions to tissue damage: some display clear signs of distress (e.g., pigs), while others (e.g., sheep) show little, if any, overt behavioural responses [1], even after surgical procedures that have been shown to be painful and aversive [2]. The assessment of pain in stoic animals such as sheep, therefore, can be particularly challenging [3,4]. Moreover, for painful husbandry practices such as tail docking, castration, ear tagging, and mulesing in lambs, the evaluation of the efficacy of pain relief may also be made difficult by the sheep’s stoic nature [5]. As such, it is clear that objective measures of pain in sheep to recognise and quantify pain are needed. 

Numerous studies have investigated the physiological and behavioural responses of lambs to common painful procedures [6,7,8,9]. However, the results of these studies have been inconsistent and sometimes contradictory, and the success of the measures in identifying and quantifying different types of potential pain-inducing scenarios, variable. For example, the adoption of abnormal standing postures in lambs following castration found by Molony et al. [10], was not evident in the research by Grant [6]. Likewise, tail wagging and kicking/foot stamping have proved useful [11,12] and not useful [6,13] for the identification of pain in lambs after castration. Furthermore, standing still (statue standing) is common in lambs following surgical castration [10,12], but not in lambs castrated with rubber rings [10], or in lambs that have been tail docked [14]. These inconsistencies pose a significant problem for the management of sheep as they may limit a producers’ motivation to use analgesics [15]. Pain relief in livestock is not only important to achieve good animal welfare, but for producers to meet societal expectations of the duty of care, which is essential to protect their social licence to farm.

It has been suggested that qualitative observation by experienced personnel is perhaps one of the best methods for capturing the complexity of pain in animals [16,17]. Indeed, qualitative assessments and observations are commonly used in veterinary fields and human medicine, with informal qualitative observations tending to accompany quantitative results within pain-related research [18]. Qualitative behavioural assessment (QBA) is a ‘whole animal’ approach that is proposed to capture the dynamic body language of animals (demeanour or behavioural expression behaviour), through the integration and summary of details of behavioural events, posture, and movement [19,20,21]. Several studies, in various livestock species, have supported QBA, indicating significant associations with standard physiological and behavioural measurements pertinent to the assessment of sheep welfare [22]. Perhaps more importantly, there is evidence demonstrating the usefulness of QBA in the study of animal pain. Indeed, QBA has been reported to identify differences in behavioural expression in animals suffering injury and presumably painful disease (skin lesions [23], mastitis [24], lameness [25], and flystrike [26]), and painful procedures [27]. The integrated, dynamic nature of this approach may capture the complex behaviour of lambs following painful routine husbandry procedures such as mulesing and tail docking.

The gold-standard for validating behavioural responses to pain involves the evaluation of responses with and without analgesic treatments [28]. The development and adoption of analgesic treatments for those procedures that cause pain in lambs, particularly mulesing (i.e., the surgical removal of strips of wool-bearing skin around the perineum and the tail to prevent flystrike, or cutaneous myiasis), has received significant attention in recent years [29,30,31,32]. Advice for the practical management of pain includes the application of the topical analgesic Tri-Solfen [33]. Further, there is evidence to suggest that the additional application of nonsteroidal anti-inflammatory drugs (NSAIDs) may provide longer and/or more pronounced pain relief in lambs [32,34,35]. The present study was part of a larger study that investigated the behavioural expression of lambs under field conditions following routine procedures performed at lamb marking (ear tagging, castration, mulesing, and tail docking), with and without the application of analgesic treatment (Tri-Solfen and an NSAID, meloxicam) [35]. We hypothesised that observers using QBA methodology would be able to differentiate between the behavioural expression of lambs undergoing painful procedures, with and without analgesia, and those that only had the procedural restraint. If the analgesic protocol provided adequate pain relief then it was expected that the lambs that received analgesia would display similar behaviour to those that had just been restrained, whereas the lambs that did not receive analgesia would display more pain-related behaviour.

## 2. Materials and Methods

The Animal and Human Ethics Committees at Murdoch University (R2903/17; R2598/13; O2780/15; 2008/021) approved this study, thus ensuring compliance with the Australian Code of Practice for the Care and Use of Animals for Scientific Purposes, the Australian Code for the Responsible Conduct of Research 2007, and the National Statement on Ethical Conduct in Human Research, 2007. The study was performed at Murdoch University’s Whitby Falls farm in Whitby, Western Australia (Latitude: −32.293056; Longitude: 116.015278) in July 2017.

### 2.1. Animals and Experimental Design

The behaviour of 30, 6–8-week-old, mixed sex (15 male and 15 female) Merino lambs was assessed from video footage collected in the paddock. The animals used in this study formed part of a larger project designed to test the efficacy of the NSAID meloxicam (Metacam 20, Ilium, Troy Laboratories Pty Ltd., Australia) in mitigating pain in lambs following ear marking, castration, tail docking, and mulesing, where other behavioural measurements were taken and other combinations of analgesics were applied [35]. In brief, the lambs used were allocated to treatments based on live-weight, sex, and rear type (number of lambs reared per ewe), and were housed in adjacent pens (50 m × 50 m) with their dams. All lambs were identified by numbers spray-painted on their sides, with additional unique marks on their head, limbs, or body. Any routine husbandry procedures: ear marking, castration using rubber rings, tail docking using a gas-heated knife, and surgical mulesing; were performed in a specially designed ‘marking cradle’ (LC264, Harvestaire Pty Ltd., Perth, Australia) by a Livestock Association of Australia accredited contractor, and all lambs (except the CONTROL group) were also vaccinated against scabby mouth (ScabiGard, Zoetis Australia Ltd., Sydney, Australia), and cheesy gland and clostridial diseases (Glanvac, Zoetis Australia Ltd., Sydney, Australia), with females also vaccinated against Johne’s disease (Gudair, Zoetis Australia Ltd., Sydney, Australia).

The 30 lambs used in the present study were randomly selected from 3 of 7 treatments imposed by Inglis, Hancock, Laurence and Thompson [35]:

(i) CONTROL lambs were held in the cradle for 60 sec but did not undergo the husbandry procedures (*n* = 10).
(ii) PLACEBO lambs were given subcutaneous saline 15 min prior to the husbandry procedures (*n* = 10).
(iii) ANALGESIC TREATED (AT) lambs were given subcutaneous meloxicam (1 mg/kg,) 15 min prior to the husbandry procedures in the cradle. This was immediately followed by application of a topical analgesic (Tri-Solfen, Lignocaine 40.6 g/L, adrenaline 24.8 mg/L, bupivacaine 4.5 g/L, cetrimide 5 g/L, Bayer Australia Ltd., Sydney, Australia) to the mulesed area and tail-docking wound (8–10 mL based on lamb weight; see Inglis, Hancock, Laurence and Thompson [35]). Analgesic agents were administered using manufacturer’s recommendations (*n* = 10).

### 2.2. Qualitative Behavioural Assessment (QBA)

#### 2.2.1. Observers and Collection of Video Footage

The QBA assessment videos were made up of 30 videos clips (one per lamb), with footage of lambs from the three experimental treatments: CONTROL, PLACEBO and AT collected in the paddock approximately 1 h 32 min (± 25 min) post procedure. Lambs were filmed at a distance (outside the bounds of the pens) using two hand-held video cameras (Panasonic HC-W570M: Panasonic Corporation, Kadoma, Japan) and footage was on average 29 ± 5 s in length. Footage was selected and edited to ensure evidence of husbandry procedures were not visible to QBA observers in either the assessment clips or in the term generation session in the Free-Choice Profiling (FCP) procedure. Consequently, observers viewed the lambs only from the front and side, so they were blind to both possible analgesic treatment and the status of the lambs regarding the husbandry procedures. However, observers were unavoidably aware of ear marking status.

Nineteen observers from Murdoch University students and staff (16 female and 3 male) were recruited to assess lamb behaviour using the FCP approach to the QBA methodology. These observers were recruited by advertising via email, flyers around the University campus, and on social media, with all those that responded accepted into the study. Of these 19 observers, only 3 (15.8%) could be classified as experienced with sheep, whereas the remaining 16 (84.2%) indicated that they had limited or no previous experience with sheep.

#### 2.2.2. Free-Choice Profiling Procedure

To complete the QBA by means of the FCP procedure, observers were required to generate their own lists of descriptive terms, and then score the behavioural expression of lambs using these. To generate terms, observers were shown a series of video clips of sheep, both lambs and adults, demonstrating a range of behaviours (*n* = 12). After watching each of these clips, observers were asked to list terms they thought described the animals’ behavioural expressions. Although observers had only 2 min after each clip to write down their terms, no limits were imposed on the number of these they could use. After all videos were watched, these lists were edited to remove terms that described actions, and for ease of scoring, negative terms were transformed to their stem word (e.g., uncomfortable became comfortable). The result was a unique, randomly ordered, list of descriptive terms for each observer. Each descriptive term in these lists was then attached to a visual analogue scale (VAS; minimum to maximum) in an electronic worksheet (Microsoft Excel 2003, North Ryde, NSW, Australia). The observers then used their own unique list of descriptive terms to score the full set of randomly ordered lamb videos (*n* = 30). With each term attached to a VAS, observers were instructed to score each animal’s behavioural expression, where their mark on the scale between minimum and maximum reflected the intensity of each animal’s expression of that term. Observers viewed the assessment clips independently and did not have the opportunity to confer with each other.

### 2.3. Statistical Analysis

For QBA, the distance from the minimum point of the VAS to where the observer had made a mark for each term was calculated (where minimum = 0 and maximum = 100), and these observer scores were analysed by means of Generalised Procrustes Analysis (GPA) (Genstat 2008, VSN International, Hemel Hempsteat, UK; Wemelsfelder et al. [19]). See Wemelsfelder et al. [19,21] for further details concerning these methods, including a detailed description of GPA analysis and output interpretation procedures.

Each of the assessment lambs received a score on the two main GPA consensus dimensions and these scores were BoxCox-transformed to conform to the requirements of parametric statistics with visual confirmation of residuals. A mixed-model analysis of variance (ANOVA) (Statistica 7.1, StatSoft-Inc., North Melbourne, Vic., Australia) was performed to determine if there was an effect of treatment (fixed factor) on the transformed GPA dimension scores given to the lambs on dimensions 1 and 2 (dependent variables), with clip ID (animal) included as a random factor. Post hoc pairwise ANOVAs comparing the GPA dimension scores between each treatment group (fixed factor) were performed with clip ID (animal) included as a random factor. To further investigate interobserver reliability, the GPA scores each lamb received from the 19 observers were correlated using Kendall’s coefficient of concordance *W*.

## 3. Results

The 19 observers using the FCP methodology generated a total of 74 unique terms to describe the lambs they were shown (average 15.2 ± 4.4 terms per observer; range 8–25). The GPA consensus profile explained 53.7% of the variation between observer scores of lambs and this differed significantly from the mean randomised profile (*t*_99_ = 60.7, *p* < 0.001). Two main dimensions of behavioural expression were identified, explaining 51.4% and 17.8% of the variation in scores given to individual lambs for GPA dimensions 1 and 2, respectively. The moderate level of agreement from the 19 observers in this group regarding the perceived behavioural expression scores of lambs is reflected by the reported *W* values of 0.66 and 0.54 on GPA dimensions 1 and 2, respectively (*p* < 0.001; Table 1). Although 4 observers fell outside the 95% confidence region, removal of these did not stop the consensus from being highly significant or alter treatment effects, nor did they appear to belong to any specific demographic group, and thus were retained in this study. 

**Figure 1:**
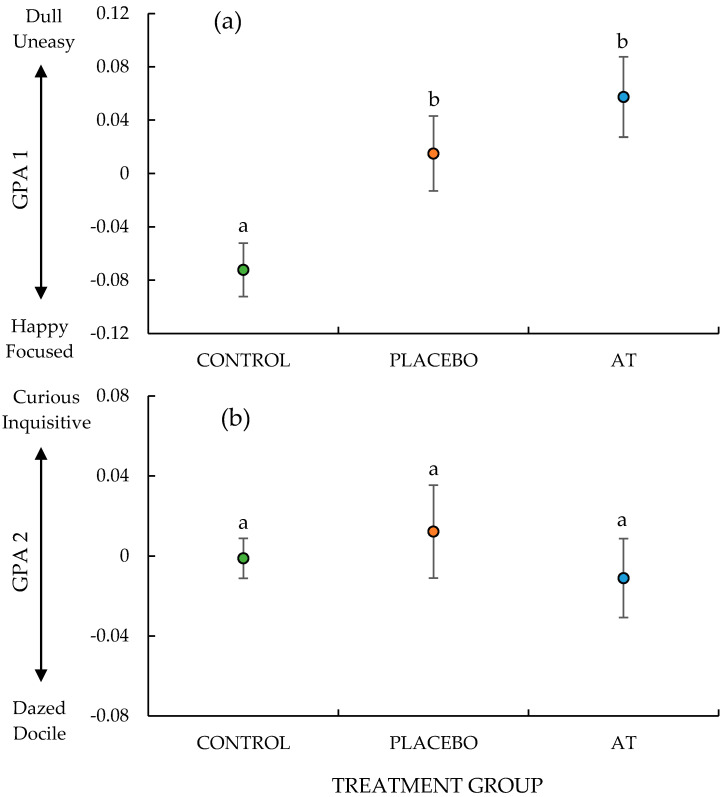
Effects of the treatment groups: CONTROL (green marker; *n* = 10), PLACEBO (orange marker; *n* = 10) and ANALGESIC TREATMENT (AT) (blue marker; *n* = 10) on General Procrustes Analysis (GPA) scores on dimension (**a**) 1 and (**b**) 2, assessed from video footage taken of lambs in the paddock approximately 1.5 h post-procedure. a and b indicate significant differences between treatment groups.

The word charts generated for each of the observers appeared to be semantically consistent, with terms converging towards similar meanings. The terms from all observers were pooled and those with the strongest loadings (positive and negative) for each of the GPA dimensions are shown in Table 1. Terms such as ‘happy’ and ‘focused’ were associated with low values for GPA dimension 1, whereas terms such as ‘dull’ and ‘uneasy’ were associated with high values on this same dimension. For GPA dimension 2, terms such as ‘dazed’ and ‘docile’ were associated with low values, and terms such as ‘curious’ and ‘inquisitive’ with high values. The two most frequently used terms for each GPA dimension, as shown in Table 1, were selected for the purpose of describing and labelling the GPA dimensions in relation to the experimental treatment group (Figure 1). 

**Figure 2:**
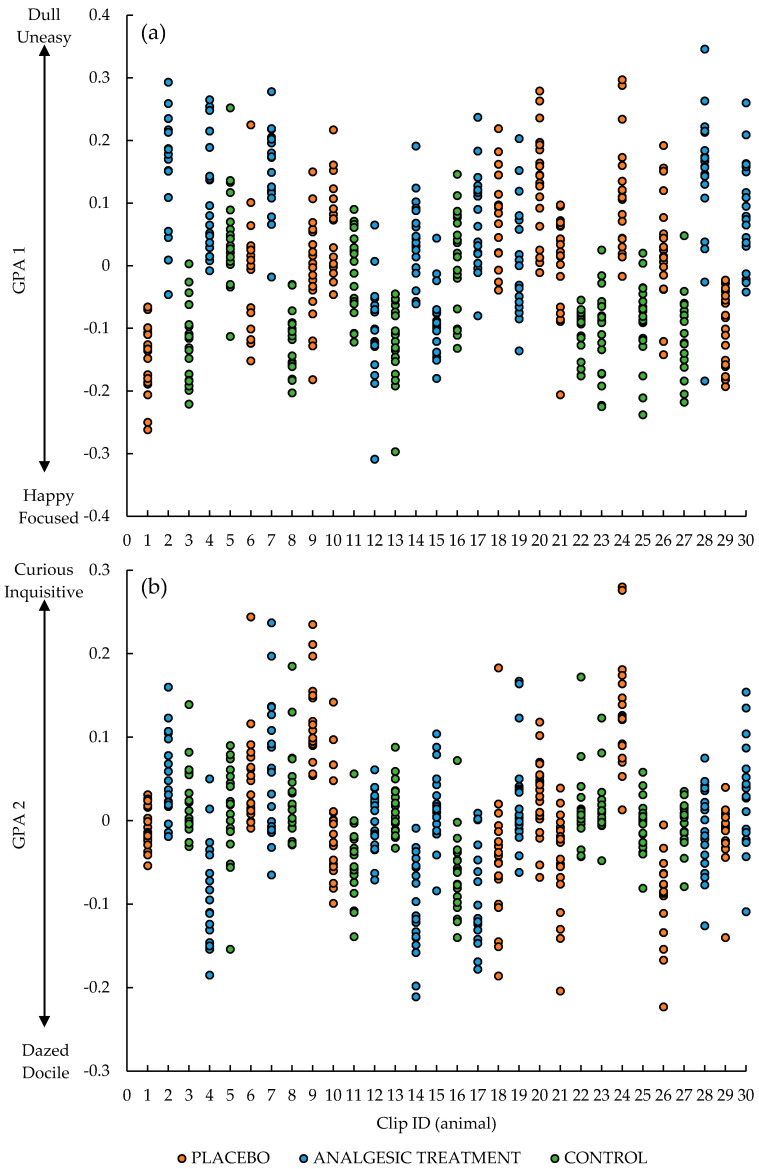
Individual observer General Procrustes Analysis (GPA) scores given to each of the 30 clips (animals) on GPA (**a**) dimension 1 and (**b**) dimension 2.

Overall, there were significant treatment effects on the first GPA dimension (F_2, 27_ = 6.25, *p* = 0.006), but not the second (F_2, 27_ = 0.39, *p* = 0.68; Figure 1). GPA dimension 1 scores for the lambs in the CONTROL treatment were significantly lower than those from the AT (F_1, 18_ = 12.82, *p* = 0.002) and the PLACEBO groups (F_1, 18_ = 6.41, *p* = 0.021), with the observers scoring the CONTROL lambs as more ‘happy’ and ‘focused’ compared to those lambs that were subject to the painful husbandry procedures both with and without analgesic treatment. There were, however, no differences between the observer scores of the lambs in the PLACEBO and AT treatments (F_1, 18_ = 1.06, *p* = 0.32) on this dimension. In addition, there were significant differences in GPA scores between clips (animals) on this dimension (F_27, 540_ = 23.43, *p* < 0.001; Figure 2) and GPA Dimension 2 (F_27, 540_ = 22.96, *p* < 0.001; Figure 2).

## 4. Discussion

To evaluate the validity of the QBA methodology to assess pain in lambs, their behavioural expression in response to routine husbandry procedures performed at lamb marking (ear tagging, castration, mulesing, and tail docking), with and without analgesic treatment, was investigated. In the present study, observers perceived a significant difference between the demeanour of CONTROL lambs and those that were subject to the painful husbandry procedures, describing the CONTROL lambs as significantly more ‘happy’ and ‘focused’ compared to both the PLACEBO and AT lambs (more ‘dull’ and ‘uneasy’). Furthermore, contrary to expectation, at 1.5 h post-procedure the behavioural expression of the lambs that received the analgesics Tri-Solfen and meloxicam (AT) was not different to that of the lambs that were given a placebo treatment with saline (PLACEBO). These results align with those of the overarching study [35] and suggest that not only do these husbandry procedures alter the behavioural expression of the lambs as expected, but that the administration of analgesics failed to normalise scores of behaviour expression in AT lambs. The latter of which implies that the analgesics provided (Tri-Solfen and meloxicam) did not ameliorate pain in lambs 1.5 h after the procedures. Given that the observers both reached a significant consensus in their assessments of behavioural expression of lambs, and identified a difference in demeanour seemingly related to the expression of pain in the AT and PLACEBO lambs, this study offers support for the use of the QBA methodology to identify the expression of pain in lambs under field conditions. Although these results are encouraging, this study represents the first step in the validation process and work is needed to verify these responses with the use of appropriate analgesics which are the gold standard [28].

In the present study, observers described and scored the behavioural expression of all animals that were subject to the painful husbandry procedures, regardless of whether they received analgesic treatment or not (AT or PLACEBO), as more ‘dull’ and ‘uneasy’ compared to the control lambs that were only restrained in the cradle for 60 s. It is undeniable that the mulesing procedure causes pain as a result of severe tissue damage and ensuing inflammatory responses, with the behavioural response to the procedure one of ‘shock’, characterised by reduced activity and the adoption of an abnormal ‘hunched’ posture while standing, with increased sensitivity to stimulation [6,30,36,37,38,39]. Furthermore, the addition of tail docking and/or castration with rubber rings at the time of mulesing in the present study is likely to have also altered the behavioural expression of lambs in a way that was evident to observers, since the combination of these procedures is known to increase the expression of active pain avoidance behaviours in lambs [6]. Thus, it is likely that the differences observed between the CONTROL lambs and the PLACEBO and AT lambs reflects the disruption of behaviour caused by these procedures. Given that the larger overarching study reported fewer normal behaviours and more pain-related behaviours in placebo lambs compared to control lambs from 1 h post-procedure, and no difference in the behaviour of the AT lambs compared to the PLACEBO and CONTROL animals until 2 h post-procedure [35], it was not unexpected that lambs subject to these painful procedures in the present study would display behavioural patterns and demeanours different to that of the control animals at 1.5 h post-procedure and that these differences would be evident to the observers using the QBA methodology. Although preliminary in nature, these results are promising and suggest that QBA may provide useful and meaningful information related to the response of lambs to severe pain caused by routine husbandry procedures performed at lamb marking. Thus, following further development and validation, QBA may become a tool to aid producers in identifying animals that are in pain, and to determine whether an animal has received sufficient analgesia, which will ultimately improve welfare.

Contrary to expectation, the administration of analgesics in the present study did not alter the behavioural expression scores between the AT and PLACEBO lambs. This lack of discernible difference in behavioural expression between the AT and PLACEBO lambs may suggest that the analgesic protocol used in the present (and the overarching) study had not yet eliminated the pain caused by the painful husbandry procedures at the time of assessment. This is an animal welfare concern that warrants urgent investigation. If this is the case, then we cannot expect our hypothesised responses (CONTROL ≠ PLACEBO, AT = CONTROL and PLACEBO ≠ AT) to hold true; rather, as was found, it is expected that AT and PLACEBO lambs would display similar behavioural responses, and that the behaviour of lambs in both of these groups would be different from that of the CONTROL lambs. Such patterns are consistent with those of previous studies in which an analgesic treatment was not effective at reducing the responses of lambs to mulesing [34,39]. Furthermore, the absence of a discernible analgesic effect on behavioural expression between AT and PLACEBO lambs at 1.5 h post-procedure in the present study align with those results of the larger overarching study, where differences in pain and lying behaviours between the equivalent AT and PLACEBO lambs only became evident from 4 h post-procedure, and differences did not appear at all in the amount of normal behaviours recorded within the first 6 h post-procedure [35]. Thus, it is likely that the analgesic protocol did not reduce the pain experienced by the AT lambs in the present study, and hence explains the lack of difference in behavioural expression between the AT and PLACEBO lambs 1.5 h post-procedure. As proposed in the overarching study [35], perhaps this lack of analgesic effect was, at least in part, a consequence of suboptimal dose rate or time of administration of meloxicam and/or Tri-Solfen. This is an issue that has also been raised by Paull, Lee, Atkinson and Fisher [39] regarding the administration of the NSAIDs meloxicam and tolfenamic acid following surgical mulesing in lambs. Alternatively, perhaps the effectiveness of the analgesic treatment to control the pain experienced by the lambs was reduced in the present (and the overarching) study since the lambs were subject to multiple procedures, and these procedures are reported to cause different types and levels of pain [6]. For example, surgical mulesing causes significant skin tissue damage, whereas castration with rubber rings causes ischaemic pain, with each procedure evoking different responses in lambs [6]. More work is needed to verify these results and to improve our understanding regarding the impact appropriate analgesic treatment has on the behavioural expression of lambs. In particular, a more detailed behavioural and physiological assessment of lambs following painful husbandry procedures together with the evaluation of the behavioural expression of lambs beyond 2 h post-procedure would prove valuable in this regard. 

The failure of the analgesic protocol to alter the behavioural expression of the AT lambs compared with the PLACEBO lambs could be considered unexpected; however, it is evident that there are inconsistencies throughout the literature concerning the onset of ameliorative effects of analgesic agents in animals following these husbandry procedures. For example, whether used singly or in combination with NSAIDs, Tri-Solfen is reported to provide rapid onset analgesia in mulesed lambs as evidenced by reductions in lamb cortisol responses, decreased behavioural indicators of pain, and significant wound anaesthesia within the first hour following mulesing and application [30,31,32]. However, there is also evidence that suggests behavioural differences between lambs mulesed with or without pain relief that incorporates Tri-Solfen and/or meloxicam (buccal or subcutaneous) only become evident from 2–4 h post-procedure [35,40]. In this situation, the assessment of animals beyond 2 h post-procedure is necessary, not simply because the verification of behavioural measures with the administration of appropriate pain relief is the gold standard for the validation of measures [28], but because it is important from a welfare perspective to confirm the efficacy and coverage that currently available analgesics offer animals. Currently, the adoption of the best-practice pain management by producers is particularly important as society expects animals raised in production industries to be treated humanely and to receive high levels of care [41]. To avoid public criticism and to protect their social licence to farm, producers must take actions to meet such expectations and this involves the application of analgesics that provide rapid and consistent amelioration of pain, particularly in instances where pain is caused by human intervention. The challenge is now to determine the most effective analgesic protocols to protect lambs from the pain caused by those routine husbandry procedures performed at marking.

The identification and assessment of pain in animals are complex [42], and may be difficult in lambs, given their behavioural responses can be subtle and conflicting [31]. Though perhaps difficult, we are obliged as a humane society to give these animals the benefit of the doubt, and in the absence of evidence that demonstrates they are not sentient, to accept that there is a moral imperative to treat them as sentient. While independent animal welfare science is critical to the debate, so too are societal morals and ethics, which shape the ‘social licence’ that permits animal use. That the observers in the present study were able to distinguish AT and PLACEBO lambs from the CONTROL lambs suggests that QBA methodology is useful in the detection of pain by allowing observers to interpret and integrate various behavioural and postural signs into a simple yet meaningful assessment. Still, it is important to note that these observed differences in the behavioural expression may not have been caused solely by the pain of the husbandry procedures the lambs underwent. For example, lambs may have had an adverse reaction to one of the vaccines administered or the ear tagging may have caused discomfort or irritation that altered or disrupted the behaviour of these lambs in a way that was perceived by the observers. Additionally, the potential impact factors such as the small sample size, inexperienced observers, and short length of observations, should also be considered in the interpretation of the results. For example, given Tri-Solfen is reported to reduce behavioural and physiological indicators of pain within 1 h post-treatment [30,31,32], it could be that the analgesic protocol administered in the overarching study provided at least some relief to the AT lambs, but that any differences in the behavioural responses of these lambs and those in the PLACEBO group were too subtle or difficult to characterise at 1.5 h post-procedure, or that the observers did not have the experience to recognise and distinguish between different pain responses, only between lambs that were in pain and those that were not. Indeed, while there is evidence that the observers’ level of experience with the focal species does not weaken QBA assessments [43,44,45], there is also evidence to the contrary [46]. There is also some evidence that observer gender may influence behavioural assessments [47] and given 84% of the observers in the present study were female, this is a factor that future studies should explore. It is also possible that the lambs were not observed for long enough for further potential differences (e.g., AT vs. PLACEBO) to become evident to observers using the QBA methodology. While there is evidence that observers can distinguish differences in sheep behavioural expression from videos of shorter duration [25,26,48,49], these studies do not explicitly focus on the assessment of pain and analgesic treatment. Indeed, the only other study involving the assessment of pain and analgesic treatment in livestock using QBA presented observers with 40–50 s of video footage per animal [27], compared to the 30 s in the present study. As such, to further validate the QBA methodology and to better understand the behavioural response of lambs to pain, efforts should be made to address these concerns. In particular, there is an obvious need to investigate the behavioural expression of a larger sample of lambs beyond 2 h post-procedure. Studies that utilise experts or at least persons more experienced than those used herein, are also necessary, particularly since those that are likely to use the approach (e.g., producers and stockpersons) are also likely to be familiar with both sheep and the assessment of pain. In addition, comparison in the use of inexperienced vs. experienced observers for the assessment of pain in lambs may be insightful. Lastly, a comparison of the behavioural expression between lambs pre- and post-surgery and analgesic treatment such as that completed by Vindevoghel, Fleming, Hyndman, Musk, Laurence and Collins [27] in their evaluation of the pain response of cattle to castration, could prove valuable. 

## 5. Conclusions

This study is the first step in investigating the validity of the QBA methodology to assess pain in lambs following those husbandry procedures performed at marking. That the observers identified differences in demeanour between CONTROL lambs and those that were subject to painful husbandry procedures and were either administered saline (PLACEBO) or analgesics (AT), strongly suggests that these procedures cause pain and that this pain alters the behavioural patterns and demeanour of lambs in a way that can be captured using the QBA methodology. Although further work is needed to verify the behavioural expression of lambs following these painful husbandry procedures, and to validate these responses to the gold standard with the use of an effective analgesic protocol, these results suggest that QBA could be a valuable tool to aid producers in the recognition and management of pain in lambs. As the results of this study and others [35,40] suggest that the use of Tri-Solfen, even in conjunction with meloxicam, may not provide effective pain relief in lambs within the first 2 h following procedures such as mulesing and tail docking that are performed at marking, it is advised that further studies are urgently needed to investigate and rectify this potential welfare gap. This study is the first of its kind in sheep and highlights several avenues for future work needed to validate QBA methodology to assess pain in this species. However, results are encouraging and demonstrate the potential for producers to use QBA to identify and manage pain in lambs. This will not only improve the welfare of lambs undergoing painful husbandry procedures, such as mulesing, but will assist the social licence for sheep farming in Australia.

## Figures and Tables

**Table 1:** Terms used by observers to describe the behavioural expression of lambs filmed in the paddock following routine painful husbandry practices (ear tagging, castration, mulesing and tail docking). Those terms that had strong loadings with the two main Generalised Procrustes Analysis (GPA) dimensions are listed. Terms with loadings >0.6 (high values) and <−0.6 (low values) are displayed for GPA dimension 1, and for GPA dimension 2, those with loadings >0.4 (high values) and <−0.4 (low values). Term order is determined first by the number of observers that used each term to assess lamb behaviour (with numbers presented in parentheses), and second by the loading of that term.

GPA Dimension (% of Variation Explained) Kendall’s *W* Score	Low Values	High Values
GPA 1 (51.4%) *W* = 0.66 ***	Happy (15), focused (8), sure (5), confident (4), motivated (1), lively (1), certain (1), at ease (1), active (1), purposeful (1), bright (1), perky (1)	Dull (5), uneasy (1), weary (1), tentative (1), sluggish (1)
GPA 2 (17.8%) *W* = 0.54 ***	Dazed (2), docile (2), secure (1), sluggish (1), weary (1)	Curious (12), inquisitive (1), lost (1), skittish (1), purposeful (1), restless (1)

*** *p* < 0.001.
